# Plasma metabolomics identifies lipid abnormalities linked to markers of inflammation, microbial translocation, and hepatic function in HIV patients receiving protease inhibitors

**DOI:** 10.1186/1471-2334-13-203

**Published:** 2013-05-04

**Authors:** Edana Cassol, Vikas Misra, Alexander Holman, Anupa Kamat, Susan Morgello, Dana Gabuzda

**Affiliations:** 1 Department of Cancer Immunology and AIDS, Dana-Farber Cancer Institute, Boston, MA, 02215, USA; 2 Department of Microbiology and Immunobiology, Harvard Medical School, Boston, MA, 02115, USA; 3 Department of Neurology, Neuroscience and Pathology, The Mount Sinai Medical Center, New York, NY, 10029, USA; 4 Department of Neurology, Harvard Medical School, Boston, MA, 02115, USA

**Keywords:** HIV, HCV, Antiretroviral therapy, Protease inhibitors, Dyslipidemia, Metabolomics, Hepatic dysfunction, Inflammation

## Abstract

**Background:**

Metabolic abnormalities are common in HIV-infected individuals on antiretroviral therapy (ART), but the biochemical details and underlying mechanisms of these disorders have not been defined.

**Methods:**

Untargeted metabolomic profiling of plasma was performed for 32 HIV patients with low nadir CD4 counts (<300 cells/ul) on protease inhibitor (PI)-based ART and 20 healthy controls using liquid or gas chromatography and mass spectrometry. Effects of Hepatitis C (HCV) co-infection and relationships between altered lipid metabolites and markers of inflammation, microbial translocation, and hepatic function were examined. Unsupervised hierarchical clustering, principal component analysis (PCA), partial least squares discriminant analysis (PLS-DA), Random forest, pathway mapping, and metabolite set enrichment analysis (MSEA) were performed using dChip, Metaboanalyst, and MSEA software.

**Results:**

A 35-metabolite signature mapping to lipid, amino acid, and nucleotide metabolism distinguished HIV patients with advanced disease on PI-based ART from controls regardless of HCV serostatus (p<0.05, false discovery rate (FDR)<0.1). Many altered lipids, including bile acids, sulfated steroids, polyunsaturated fatty acids, and eicosanoids, were ligands of nuclear receptors that regulate metabolism and inflammation. Distinct clusters of altered lipids correlated with markers of inflammation (interferon-α and interleukin-6), microbial translocation (lipopolysaccharide (LPS) and LPS-binding protein), and hepatic function (bilirubin) (p<0.05). Lipid alterations showed substantial overlap with those reported in non-alcoholic fatty liver disease (NALFD). Increased bile acids were associated with noninvasive markers of hepatic fibrosis (FIB-4, APRI, and YKL-40) and correlated with acylcarnitines, a marker of mitochondrial dysfunction.

**Conclusions:**

Lipid alterations in HIV patients receiving PI-based ART are linked to markers of inflammation, microbial translocation, and hepatic function, suggesting that therapeutic strategies attenuating dysregulated innate immune activation and hepatic dysfunction may be beneficial for prevention and treatment of metabolic disorders in HIV patients.

## Background

Despite the success of combination antiretroviral therapy (ART) in reducing HIV-associated morbidity and mortality, long-term ART is frequently associated with metabolic abnormalities including dyslipidemia, lipodystrophy, and insulin resistance [1,2]. These metabolic abnormalities increase the risk of cardiovascular, liver, kidney, bone, and neurological disorders and the incidence of these disorders is rising as HIV-infected populations age [1,3]. Mechanisms driving these abnormalities are multifactorial including effects of ART (e.g., protease inhibitor (PI)-associated dyslipidemia and nucleoside reverse transcriptase inhibitor (NRTI)-associated mitochondrial toxicity [2-4]), disease related factors (e.g., CD4 T-cell depletion, inflammation, and unsuppressed viremia), and host factors (e.g. body mass index, comorbidities, and genetic predisposition) [1,2].

The liver plays a central role in regulating lipid, amino acid, and carbohydrate metabolism, but few studies have explored relationships between hepatic dysfunction and metabolic abnormalities in HIV-infected individuals on ART. Liver disease represents a leading cause of morbidity and mortality in HIV patients on ART, with hepatic dysfunction affecting 30-40% of patients [5-7]. Ranging from mild reversible increases in hepatic enzymes to fibrosis and decompensation, hepatic dysfunction has been linked to co-infections with hepatitis B and C (HBV and HCV), ART-induced hepatotoxicity, and high prevalence of non-alcoholic fatty liver disease (NAFLD), which affects 20-70% of HIV-infected individuals [5].

Twenty-five to 40% of HIV-infected individuals in the United States and Europe are co-infected with HCV. In these populations, HCV co-infection is associated with increased rates of lipodystrophy [8,9], hepatic steatosis [10-12], and insulin resistance [9,13,14], but lower total cholesterol (TC) and low-density lipoprotein cholesterol (LDL) [15-17], particularly in patients infected with HCV genotype 3 [11,12,18]. In HCV mono-infected individuals, altered cholesterol metabolism is associated with hepatic steatosis, advanced hepatic fibrosis, and poor responses to interferon-based therapy [19-21]. Further studies are required to better define metabolic alterations in HIV subjects with and without HCV co-infection.

Metabolomics is the unbiased identification and quantification of small molecules in biological fluids. In the context of disease, metabolomics has been used to identify novel clinical biomarkers and therapeutic targets. Here, we performed untargeted metabolomic profiling of plasma from two independent cohorts of HIV-infected individuals with late stage disease on PI-based ART to identify a metabolite signature that distinguishes HIV-infected from healthy control subjects regardless of HCV serostatus. We also examined relationships between altered lipid metabolites and markers of inflammation, microbial translocation, and hepatic dysfunction.

## Methods

### Study subjects

HIV subjects (n=32) in the two independent cohorts were from the National NeuroAIDS Tissue Consortium (NNTC) (Manhattan HIV Brain Bank, National Neurological AIDS Bank, California NeuroAIDS Tissue Network, Texas NeuroAIDS Research Center) and CNS HIV Anti-Retroviral Therapy Effects Research (CHARTER) study. Subjects were enrolled with written informed consent and IRB approval at each study site. Inclusion criteria were advanced disease (nadir CD4<300 cells/ul), HIV plasma viral load <400 copies/ml, and >1 year on PI-based ART (31% receiving lopinavir (LPV) plus ritonavir (RTV), 22% receiving nelfinavir (NFV), 16% receiving saquinavir (SQV) plus RTV, 10% receiving atazanavir (ATV) plus RTV, 6% receiving fosamprenavir (FPV) plus RTV, 6% receiving indinavir (IDV) plus RTV, 6% receiving SQV and NFV plus RTV, and 3% receiving amprenavir). Exclusion criteria were current use of lipid lowering medications, current heavy alcohol consumption, severe hepatotoxicity (defined in accordance with the AIDS Clinical Trials Group criteria as grades 3 or 4 [22]), and moderate to severe renal insufficiency [23]. To evaluate effects of HCV co-infection (defined by positive serostatus), the initial cohort included HIV subjects with and without HCV co-infection (n=17). Results were validated in a second independent cohort of HIV subjects co-infected with HCV (n=15; defined by serostatus). HIV subjects in the initial and validation cohorts were matched for age, gender, race/ethnicity, and stage of HIV disease (nadir and current CD4 counts). Results were also validated in an independent cohort of HIV subjects on non-nucleoside reverse transcriptase inhibitors (NNRTI)-based ART (88% on efavirenz and 22% on nevirapine) matched to HIV subjects on PI-based ART in the initial and validation cohorts by age, gender, HCV serostatus, and stage of HIV disease (nadir and current CD4 counts). Healthy control samples were HIV- and HCV-negative individuals without a diagnosis of diabetes or hyperlipidemia obtained from Bioreclamation (Westbury, New York) and with informed consent and IRB approval from Dana-Farber Cancer Institute. Controls were matched by gender and by gender, age, race/ethnicity, and smoking in the initial and validation cohorts, respectively.

### Quantification of soluble markers

Plasma levels of interferon (IFN)-α, interleukin (IL)-6, and IL-2 receptor alpha (RA) were measured using a Luminex multiplex assay (Bio-Plex System, Bio-Rad Laboratories). Plasma lipopolysaccaride (LPS) was quantified as previously described [24]. Soluble CD14 (sCD14) (R&D Systems), LPS-binding protein (LBP) (Cell Sciences), and YKL-40 (Quidel Corporation) were quantified by ELISA.

### Metabolomic profiling

Metabolomic profiling was performed by Metabolon (Durham, NC) using ultra high performance liquid chromatography and tandem mass spectrometry (UHLC/MS^2^/MS) optimized for the detection of acidic or basic metabolites, and gas chromatography (GC)/MS. Plasma samples were extracted using the MicroLab STAR system as described [25]. The UHLC/MS^2^/MS platform was based on a Waters ACQUITY UHPLC and Thermo-Finnigan LTQ mass spectrometer, which consisted of an electrospray ionization source and linear ion-trap mass analyzer. Derivatized samples for GC/MS were separated on 5% phenyldimethyl silicone column with helium as the carrier gas and a temperature ramp from 60° to 340°C over a 16 minute period. Analysis was performed on a Thermo-Finnigan Trace DSQ fast-scanning single-quadrupole mass spectrometer using electron impact ionization. Compounds were identified by automated comparison of chromatographic and mass spectra properties in samples to metabolomic library entries of purified standards.

### Data processing, bioinformatics, and statistical analysis

Metabolite data was normalized by median centering. Missing values (33% of metabolites had at least one missing value) were imputed with the lower limit of detection for a given metabolite. Batch normalization was performed using the median ratio for each metabolite in duplicate “anchor” samples across runs. Significantly altered metabolites were defined by a fold change (FC) >1.3, p-value <0.05 and FDR ≤0.1. Principal component analysis (PCA), partial least squares discriminant analysis (PLS-DA), and Random Forest were performed in the Metaboanalyst web portal (http://www.metaboanalyst.ca). Hierarchical clustering of signature metabolites altered in HIV compared to control subjects was performed in dChip (http://www.dchip.org). KEGG API was used to interface with the KEGG database. Additional small molecule pathways were extracted from Small Molecule Pathways Database (SMPDB). Quantitative enrichment analysis was performed in Metabolite Set Enrichment Analysis (MSEA) software (http://www.metaboanalyst.ca) using the Metabolic Pathways library from MSEA and metabolite sets from Lipid Maps (http://www.lipidmaps.org). Additional statistical analyses were performed in R on log-transformed data. Pearson correlations were used to evaluate relationships between metabolites (p<0.05, FDR≤0.1). Spearman correlations were used to examine relationships between metabolites and markers of inflammation (IFN-α, IL-6, IL2RA, and sCD14), microbial translocation (LPS and LBP), and hepatic function (total bilirubin, bilirubin (E,E), bilirubin (Z,Z), and albumin), and liver enzymes (alanine transaminase (ALT), aspartate transaminase (AST), and alkaline phosphatase (ALP))(p<0.05, FDR≤0.1). Multiple testing corrections were performed by calculating the false discovery rate (FDR) using fdrtool.

## Results

### HIV subject cohorts

Clinical characteristics of HIV subjects in the two cohorts are shown in Table 1. Both cohorts were predominantly male and African American (median age 45), with similar nadir and current CD4 T cell counts (median 58 vs. 53 (p=0.314) and 267 vs. 212 cells/ul (p=0.472)) and suppressed plasma viral loads (median 69 vs. 50 copies/ml) in initial and validation cohorts, respectively. All HIV subjects were on ART including at least one protease inhibitor for a median of 31 months (IQR: 15–50 months); 75% were on RTV-boosted PI and 25% were on unboosted PI. 97% of HIV subjects were taking NRTI inhibitors. Only three HIV subjects had hyperlipidemia (2 with mildly elevated total cholesterol, LDL, and triglycerides, 1 with high triglycerides and mildly elevated total cholesterol), one subject had clinical evidence of lipodystrophy, and one had diabetes. 38% of HIV subjects had mild-to-moderate elevations in hepatic aminotransferases (ALT range: 62–113 IU/L and AST range: 38–133 IU/L) and 19% had elevations in total bilirubin (range: 1.3-4.2 mg/dL) and/or decreased albumin (range: 2–3.3 g/dL). HCV co-infection was not associated with higher levels of ALT, AST, or total bilirubin compared to those without HCV co-infection. 31% of HIV subjects reported past alcohol abuse and one had current alcohol dependence (assessed by Psychiatric Research Interview for Substance and Mental Disorders (PRISM)).

**Table 1:** Clinical and demographic characteristics of HIV subjects in study cohorts

**Characteristic**	**Initial cohort (n=17)**	**Validation cohort (n=15)**
Age (years)*	45 (42–50)	45 (43–50)
Gender (male)	12 (60%)	15 (83%)
Race (African American)	15 (75%)	9 (50%)
BMI	23.7 (22.9-24.7)	23.9 (22.7-27.2)
HCV seropositive	7 (41%)	15 (100%)
Time on ART (months)*	30 (13–50)	36 (16–50)
CD4 T-cell count (cells/ul)*	267 (107–399)	213 (126–293)
Nadir CD4 T-cell count (cells/ul)*	58 (18–136)	53 (9–132)
Plasma HIV RNA (copies/ml)*	69 (50–181)	50 (50–88)
Total Bilirubin (mg/dL)*	0.6 (0.4-0.9)	0.6 (0.4-0.9)
ALT (IU/L)*	31 (23–77)	27 (20–38)
AST (IU/L)*	36 (25–59)	33 (24–52)
Alkaline Phosphatase (IU/L)*	96 (81–128)	88 (80–128)
Albumin (g/dL)*	4.0 (3.7-4.5)	4.0 (3.7-4.4)

* Median (IQR).

### Alterations in the plasma metabolome of HIV subjects are linked to multiple biosynthetic pathways

**Figure 1:**
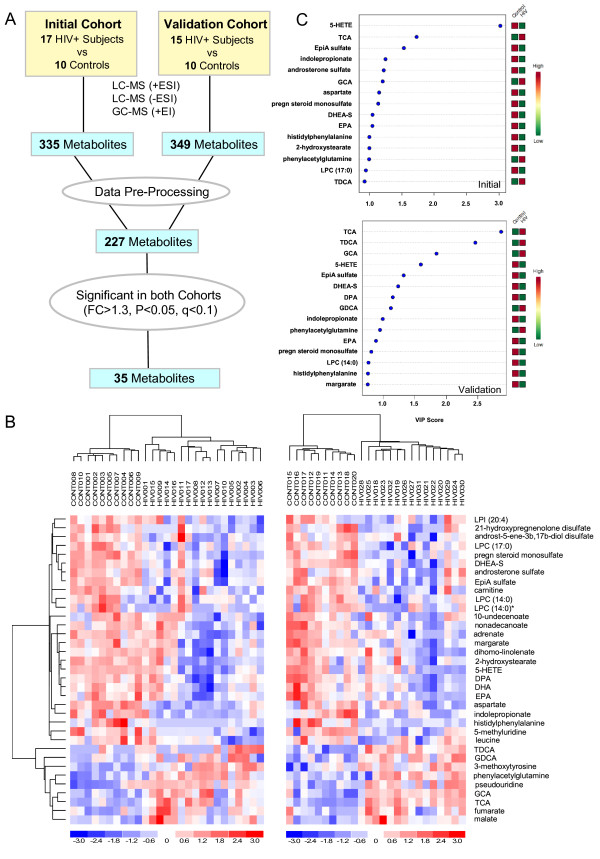
**Metabolomic profiling of plasma from two cohorts identifies metabolites that distinguish HIV subjects from controls.** (**A**) Schematic of strategy used to identify plasma metabolites altered in initial and validation cohorts. LC, liquid chromatography; GC, gas chromatography; MS, mass spectrometry; ESI, electrospray ionization; EI, electron ionization. (**B**) Unsupervised hierarchical clustering of signature metabolites (n=35) that distinguish HIV vs. control subjects in initial and validation cohorts (left and right heatmaps, respectively) regardless of HCV serostatus (FC>1.3, p<0.05, FDR <10%). Red and blue indicate increased and decreased levels, respectively. (**C**) Top VIP scores with expression heatmap from PLS-DA models. PLS-DA models were constructed with signature metabolites (n=35) from the initial and validation cohorts. Red and green indicate increased and decreased levels, respectively.

**Figure 2:**
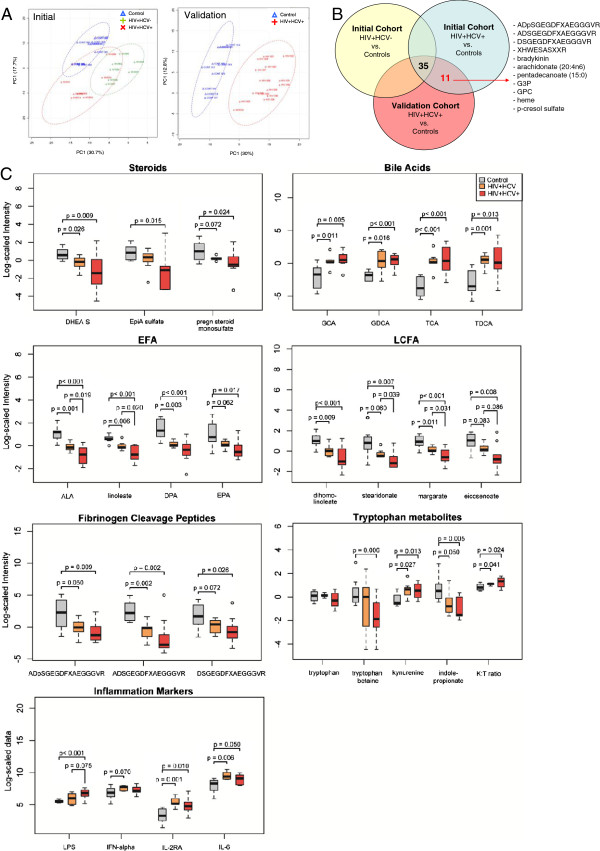
**Metabolic pathways altered in HIV**^**+**^**HCV**^**- **^**and HIV**^**+**^**HCV**^**+ **^**subjects on PI-based ART.** (**A**) PCA analysis shows separation of control (blue), HIV^+^HCV^-^ (green), and HIV^+^HCV^+^ (red) metabolomes (n=227 metabolites) in initial and validation cohorts. (**B**) Venn diagram showing the distribution of metabolites altered in HIV^+^HCV^+^ subjects in initial and validation cohorts (n=46; FC>1.3, p<0.05, FDR<10%). (**C**) Box plots of metabolite classes altered in HIV^+^HCV^-^ (n=7) or HIV^+^HCV^+^ (n=10) subjects compared to controls (n=8) matched for age, gender, and race/ethnicity. Medians are represented by horizontal bars, boxes span the interquartile range (IQR) and whiskers extend to extreme data points within 1.5 times IQR. Outliers plotted as open circles lie outside 1.5 times the IQR. Grey, orange, and red box plots represent controls, HIV^+^HCV^-^ and HIV^+^HCV^+^ subjects, respectively. P-values were calculated using Welch’s t-tests. K:T ratio; kynurenine to tryptophan ratio.

Untargeted metabolomic profiling of plasma from 2 independent cohorts of HIV and control subjects detected a total of 335 and 349 metabolites in the initial and validation cohorts, respectively (Figure 1A). To reduce noise in the data analysis, pre-processing was performed to exclude xenobiotics, carbohydrates, and metabolites >3-fold different between the control groups (p<0.01). By this approach, 227 metabolites detected across both cohorts met acceptability criteria and were further analyzed using bioinformatic tools (Additional file 1). PCA revealed a clear separation of HIV and control metabolomes in both cohorts (Figure 2A). Consistent with these results, Random Forest analysis classified HIV vs. control subjects with 96% and 100% predictive accuracy in initial and validation cohorts, respectively. 35 metabolites distinguished between HIV subjects and controls across both cohorts (10 up and 26 down) regardless of HCV serostatus based fold change analysis (FC>1.3, p<0.05, FDR<10%) and subsequent sub-analysis of HIV subjects from the initial cohort with and without HCV co-infection. Moreover, the majority of these metabolites (23 of 35), including bile acids, steroids, and long chain fatty acids (LCFA), were altered in an independent cohort of HIV subjects on NNRTI-based ART vs. matched healthy controls (Additional file 2), indicating that many of these alterations also occur in HIV patients not on PI-based ART. Unsupervised hierarchical clustering of this metabolite set classified HIV vs. control subjects with 100% accuracy in both cohorts (Figure 1B). To identify and rank signature metabolites explaining most of the variance between HIV and control metabolomes, we used PLS-DA to evaluate variable importance in projection scores (VIP scores) (Figure 1C). Eleven of 15 top-ranked VIP scores overlapped between cohorts (p<0.05), corresponding to 3 bile acids (glycocholate (GCA), taurocholate (TCA), and taurodeoxycholate (TDCA)), 3 steroids (dehydroepiandrosterone sulfate (DHEA-S), epiandrosterone sulfate (EpiA-S), and pregnenolone sulfate), eicosapentaenoic acid (EPA), 5-hydroxyeicosatetraenoic acid (5-HETE), indolepropionate, phenylacetylglutamine, and histidylphenylalanine. Thus, we identified a 35-metabolite signature that distinguishes HIV-infected from healthy control subjects regardless of HCV serostatus.

### Substantial overlap between alterations in the plasma metabolome of HIV subjects with and without HCV co-infection

Given the high prevalence of HCV co-infection in HIV-infected populations and the association between HCV co-infection and increased rates of lipodystrophy, hepatic steatosis, and insulin resistance, we next examined the effects of HCV co-infection on the HIV plasma metabolome. Within the initial cohort, PCA revealed a clear separation of the plasma metabolomes from HIV subjects with and without HCV co-infection (Figure 2A). To examine the effects of HCV co-infection on the HIV plasma metabolome, we compared metabolic alterations in subgroups consisting of HIV subjects with and without HCV co-infection (Figure 2B). In addition to the 35-metabolite signature that distinguished HIV subjects from healthy controls regardless of HCV serostatus (Figure 1B), 11 additional metabolites (2 up and 9 down) (FC>1.3, p<0.05, FDR<10%) were altered only in HCV-positive subjects across both cohorts (Figure 2B). Next, we performed a sub-analysis of HIV subjects with (n=10) and without HCV co-infection (n=7) and healthy controls (n=8) matched for age, gender, and race/ethnicity. The majority of metabolites altered in HIV subjects with HCV co-infection were also altered in subjects without HCV including sulfated steroids, bile acids, essential fatty acids (EFA), LCFA, fibrinogen cleavage peptides, and tryptophan metabolites (Figure 2C and Additional file 3). Compared to HIV subjects without HCV, HCV co-infection was associated with lower sulfated steroids, essential fatty acids (EFA), long chain fatty acids (LCFA), fibrinogen cleavage peptides, and tryptophan metabolites (i.e. tryptophan betaine and indolepropionate) and higher plasma LPS (Figure 2C). Unexpectedly, bile acid levels were similar in HIV subjects with and without HCV. These results suggest there is substantial overlap between metabolite sets altered in HIV subjects with versus without HCV co-infection in the study cohort. However, HCV co-infection appears to potentiate some of these metabolic abnormalities.

### Altered bile acid, acylcarnitine, PUFA, glycerophospholipid, and steroid metabolism in HIV subjects on PI-based ART

**Figure 3:**
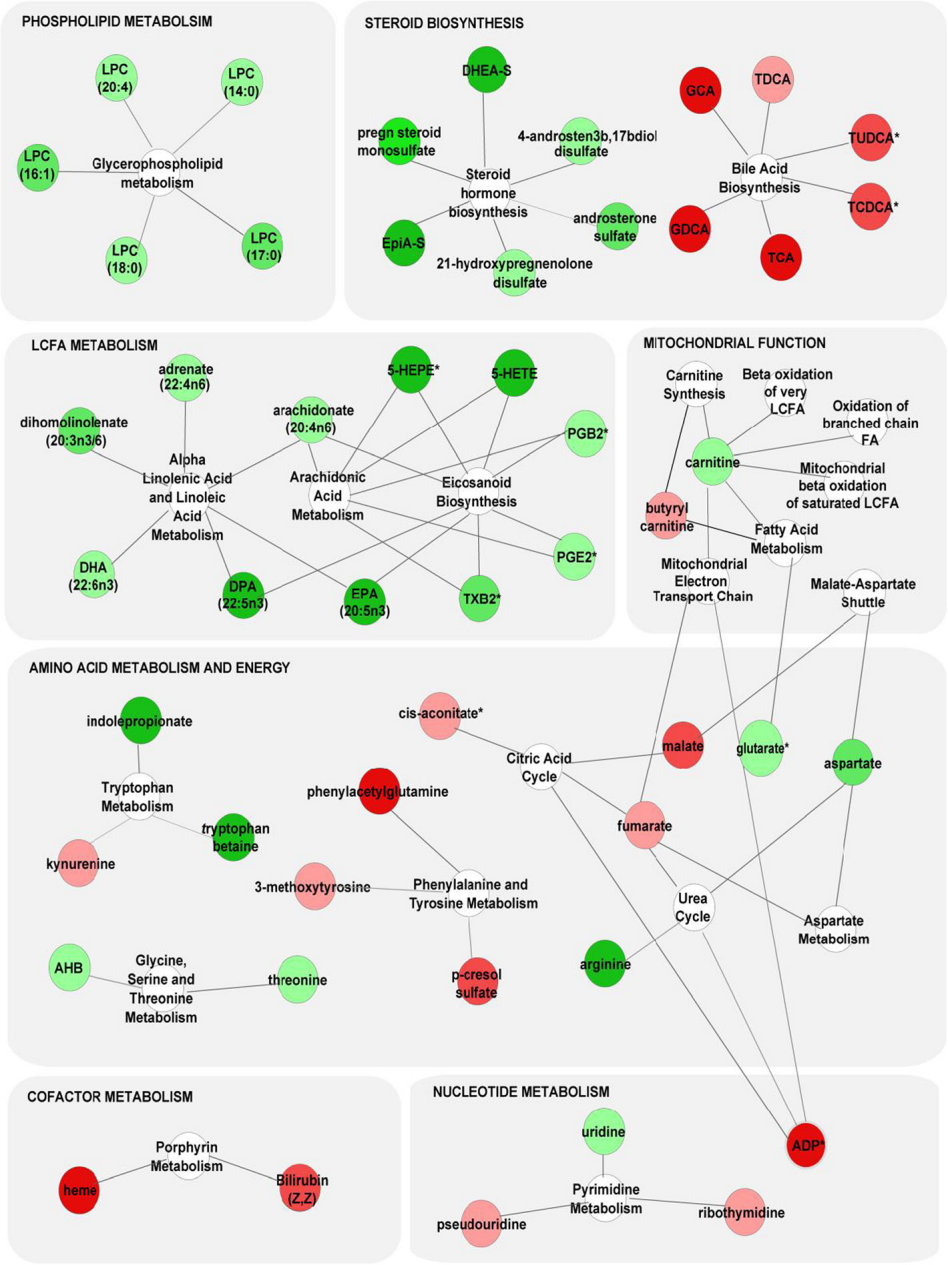
**Metabolites altered in HIV subjects compared to healthy controls map to multiple biosynthetic pathways.** Altered metabolites with KEGG ID from the merged dataset (n=32 HIV and n=20 control subjects; FC>1.3, p<0.05, FDR<0.1) were mapped to KEGG and SMPDB reference pathways and interaction networks were generated in Cytoscape. Green and red nodes represent metabolites with increased and decreased levels, respectively. White nodes represent pathways. Asterisks indicate related metabolites detected in only one cohort.

**Figure 4:**
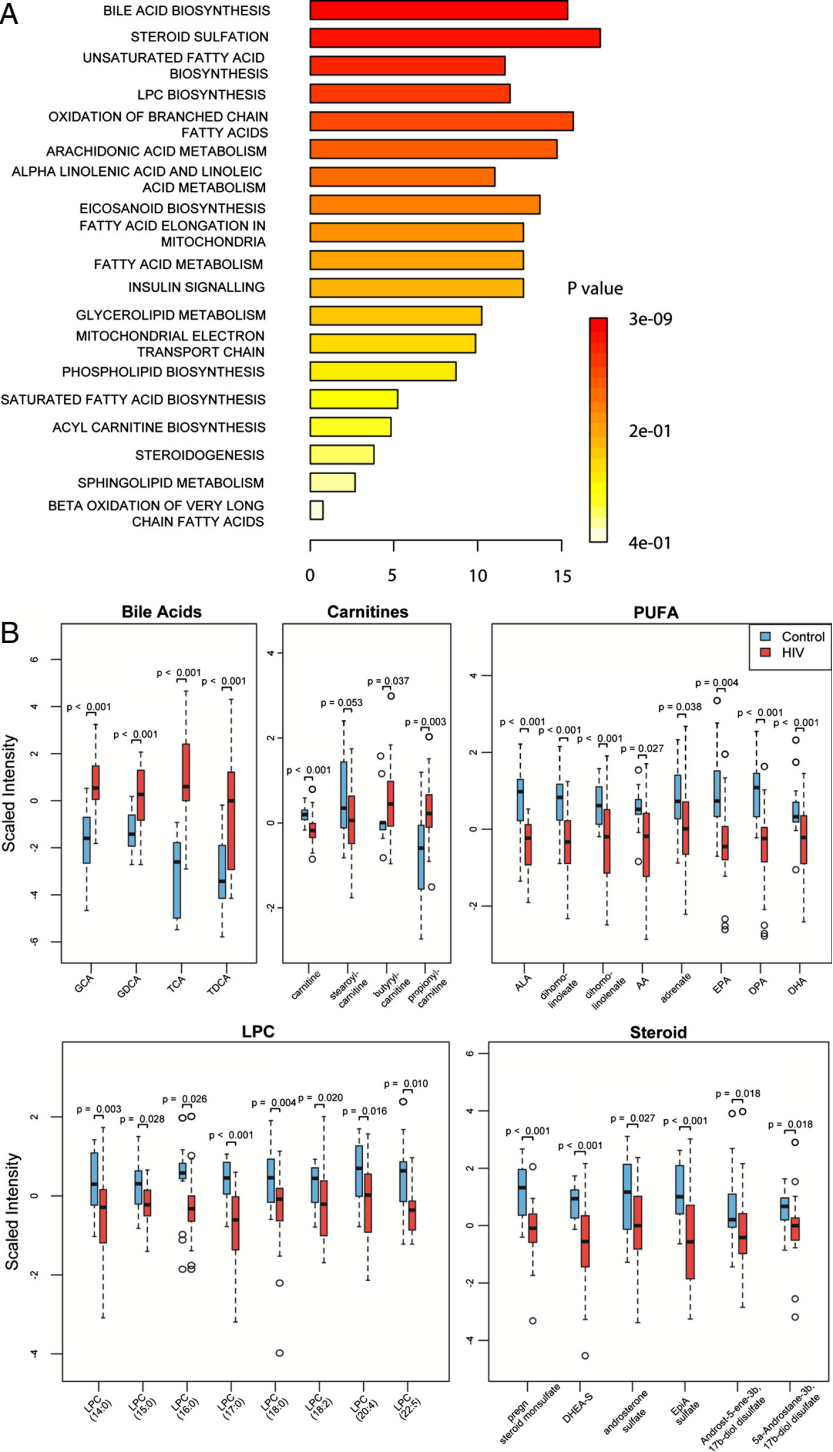
**Lipid pathways altered in HIV subjects on PI-based ART.** (**A**) Quantitative Enrichment Analysis (QEA) performed using MSEA. QEA is based on the globaltest algorithm which uses a generalized linear model to estimate a Q-statistic for each metabolite set. Lipid metabolites (n=113) from the merged dataset consisting of all HIV subjects (n=32) and controls (n=20) were inputted into MSEA and enrichment was assessed using the MSEA Metabolic Pathway library (n=88) and custom metabolite sets derived from Lipid Maps (n=5). Pathways were considered enriched when p<0.05 and FDR<5%. (**B**) Box plots of major lipid classes altered in HIV subjects (n=32) compared to controls (n=20). Medians are represented by horizontal bars, boxes span the interquartile range (IQR) and whiskers extend to extreme data points within 1.5 times IQR. Outliers plotted as open circles lie outside 1.5 times the IQR. Blue and red represent controls and HIV subjects, respectively. P-values were calculated using Welch’s t-tests.

Given substantial overlap between metabolites altered in HIV subjects with and without HCV co-infection, we used a merged dataset of all HIV subjects (n=32) and healthy controls (n=20) for pathway mapping and correlation analysis. In the merged dataset, 58 metabolites distinguished between HIV subjects and controls (FC>1.3, p<0.05, FDR<10%), including the 35 signature metabolites and 23 additional metabolites that approached significance in the initial and validation cohorts (0.05<p<0.20). Mapping these metabolites with KEGG IDs (n=50) to KEGG and SMPDB pathways identified alterations in multiple biosynthetic pathways including glycerophospholipid metabolism (lysophosphocholine (LPC)), steroid metabolism (pregnenolone sulfate, DHEA-S, EpiA-S, and androsterone sulfate), bile acid biosynthesis (TCA, taurodeoxycholate (TCDA), GCA, and glycodeoxycholate (GCDA)), LCFA metabolism (arachidonic acid (AA), EPA, docosahexaenoic acid (DHA), and docosapentaenoic acid (DPA)), eicosanoid biosynthesis (5-HETE, prostaglandin B2 (PGB2), prostaglandin E2 (PGE2), and thromboxane B2 (TXB2)), mitochondrial function (carnitine and butyrylcarnitine), tryptophan metabolism (tryptophan betaine, kynurenine, and indoleproprionate), the citric acid cycle (fumarate and malate), and pyrimidine metabolism (uridine, pseudouridine, and 5-methyluridine) (Figure 3). Because >50% of altered metabolites were lipids, we performed MSEA for 113 lipids from the merged dataset (Additional file 4). 11 metabolite sets were enriched in the HIV plasma lipidome compared to controls (p<0.05, FDR<5%) including bile acid biosynthesis, steroidogenesis and sulfation, LPC and acylcarnitine biosynthesis, and pathways involved in fatty acid biosynthesis, elongation, oxidation, and metabolism (Figure 4A). Consistent with these results, Welch’s t-tests identified alterations in several lipid classes including bile acids, acylcarnitines, PUFA, LPC, and sulfated steroids (FC>1.3, p<0.05, FDR<10%) (Figure 4B). Four bile acids and two acylcarnitines were increased and omega-3 and omega-6 PUFA (AA, EPA, DPA, and DHA), LPC ((LPC (18:0), LPC (18:1), and LPC (20:4)), and sulfated steroids (DHEA-S, pregnenolone sulfate, androsterone sulfate and EpiA-S) were decreased in HIV compared to control subjects. Thus, alterations in the HIV plasma metabolome are indicative of dysregulated of bile acid, acylcarnitine, PUFA, LPC, and steroid metabolism.

### Identification of lipid abnormalities linked to markers of inflammation, microbial translocation, and hepatic function

**Figure 5:**
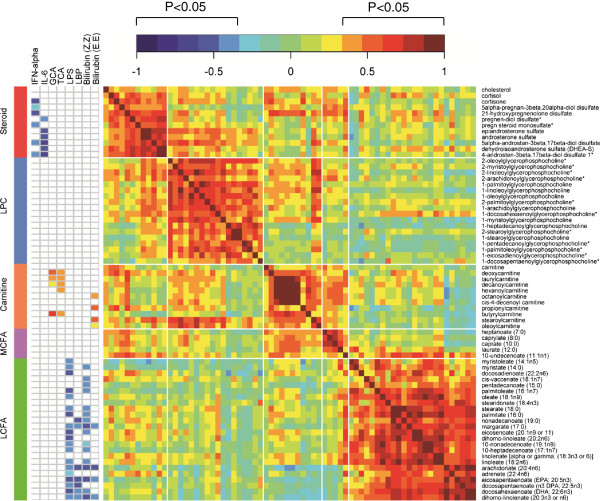
**Correlation matrix reveals relationships between clusters of lipids and markers of inflammation and hepatic function.** The Pearson correlation matrix was constructed in R using the heatmap.2 function. Significant correlations had a −0.35> r >0.35, p<0.05, and FDR<10% (Additional file 5). Red and blue indicate positive and negative correlations, respectively (see Color Key).

Next, we examined inter-relationships between alterations in the plasma lipidome and markers of inflammation, microbial translocation, and hepatic function. Pearson correlation analysis followed by clustering and visualization of the correlation matrix identified 3 distinct clusters of co-modulated lipids (p<0.05, FDR<0.1) (Figure 5, Additional file 5). In cluster 1, steroids and LPC were positively correlated. In cluster 2, acylcarnitines and medium chain fatty acids were positively correlated. In cluster 3, LCFA were positively correlated. Next, we examined correlations between these lipid subsets and markers of inflammation (IFN-α, IL-6, IL-2RA, and sCD14), microbial translocation (LPS, and LBP), hepatic function (bilirubin and albumin), and liver enzymes (ALT, AST, and ALP). In cluster 1, sulfated steroids correlated negatively with IFN-α and IL-6 (p<0.05, FDR<10%) (Figure 5). In cluster 2, acylcarnitines correlated positively with bile acids (GCA and TCA) and bilirubin (E,E), a geometric isomer of bilirubin identified by metabolomic profiling. In Cluster 3, LCFA correlated negatively with LPS, LBP, and bilirubin (Z,Z) and (E,E). LPS and LBP are markers of microbial translocation from a damaged gut that have been previously linked to chronic innate immune activation [24,26] and hepatic dysfunction (9–10) in HIV infection. These results identify distinct alterations in the HIV plasma lipidome linked to markers of inflammation, microbial translocation, and hepatic function.

### Overlap between alterations in the HIV plasma lipidome and changes previously described in NAFLD/Nonalcoholic Steatohepatitis (NASH)

**Figure 6:**
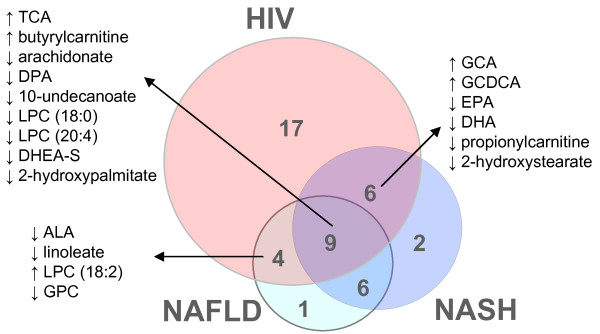
**The HIV plasma lipidome has overlapping yet distinctive features compared to alterations in NAFLD/NASH.** Venn diagram depicting overlap between metabolites altered in the same direction in HIV subjects on PI-based ART and HIV-negative subjects with NAFLD or NASH (n=45, Additional file 6). NAFLD and NASH data sets were based on 3 published studies [27-29].

Given the high prevalence of NAFLD/NASH in HIV patients on ART [6,7], we compared alterations in the HIV plasma lipidome to those previously reported in 3 independent studies of HIV-negative patients with NAFLD/NASH [27-29]. Of 113 lipids detected in plasma, 60 were previously studied in subjects with NAFLD/NASH and 45 were altered in HIV or NAFLD/NASH. 53% (19/36) of lipids altered in HIV subjects overlapped with those altered in NAFLD/NASH, including increased bile acids and acylcarnitines and decreased LPC, PUFA, and DHEA-S (Figure 6, Additional file 6). However, 47% (17/36) of altered lipids were unique to HIV subjects, including decreased saturated LCFA and eicosanoids. Thus, the plasma lipidome of HIV patients on suppressive ART has overlapping, yet distinctive features compared to those reported in NAFLD/NASH.

### Increased plasma bile acids are associated with noninvasive markers of liver fibrosis (FIB-4, APRI, and YKL-40)

**Figure 7:**
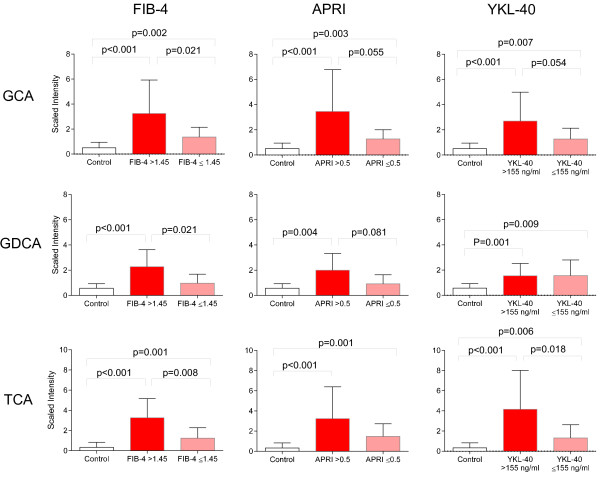
**Increased plasma bile acids are associated with intermediate/high FIB-4 and APRI scores and high YKL-40.** Bar graphs showing bile acid levels (scaled intensity) in healthy controls (white), and HIV subjects with low (pink) vs. intermediate/high FIB-4 or APRI scores or high YKL-40 levels (red). P-values were calculated using Welch’s t-tests.

Recent studies suggest serum bile acids may represent a noninvasive marker of liver disease in patients with HCV [30]. To explore relationships between bile acids and non-invasive markers of liver fibrosis in HIV patients on ART, we calculated FIB-4 (age [years] × AST [IU/L]/platelet count [10^9^/L] × (ALT^1/2^[IU/L])) and AST-Platelets Ratio Index (APRI) (AST [IU/L]/platelet count [10^9^/L]) scores, evaluated YKL-40 levels in plasma, and examined associations between these markers and alterations in bile acids. FIB-4, APRI, and YKL-40 are non-invasive markers of liver fibrosis shown to correlate with the Ishak histology scoring system [31-37]. Using previously described cut-offs [31,38], 44 and 47% of HIV subjects had intermediate to high FIB-4 (>1.45) and APRI (>0.5) scores, respectively. 53% of HIV subjects had high (>155 ng/ml) levels of plasma YKL-40. HIV subjects with intermediate/high FIB-4 and APRI scores had higher plasma GCA, GDCA, and TCA compared to those with low scores and controls (Figure 7). Consistent with these findings, HIV subjects with high levels of circulating YKL-40 had higher levels of plasma GCA and TCA. These results suggest that elevated plasma bile acids are associated with non-invasive markers of hepatic fibrosis in HIV patients with advanced disease on PI-based ART.

## Discussion

In this study, we characterized the plasma metabolome of HIV patients with advanced disease (CD4 nadir <300 cells/ul) on PI-based ART and identified a 35-metabolite signature mapping to lipid, amino acid, and nucleotide metabolism that distinguished HIV from control subjects independent of HCV serostatus. Seventy percent of these altered metabolites were lipids, including endogenous ligands for the nuclear receptors FXR, PXR, CAR, and PPAR such as bile acids, sulfated steroids, and PUFA. These receptors play important roles in regulating lipid metabolism, inflammation, and hepatic function. Lipids regulate diverse cellular processes including metabolic, inflammatory, and innate immune responses, and imbalances in lipid signaling pathways contribute to diverse pathologies including insulin resistance and cardiovascular disease [39-41]. Untargeted metabolomic profiling represents a powerful tool for deconvoluting these pathways and gaining insights important for developing therapeutic strategies to reduce metabolic disease and chronic inflammation in HIV patients on ART.

In treatment-naive HIV patients studied prior to the introduction of ART, dyslipidemia was frequently detected, and characterized by hypocholesterolemia with and without hypertriglyceridemia [42,43]. The severity and frequency of these abnormalities were associated with decreasing CD4 counts and advanced disease [43,44]. In HIV patients on ART, dyslipidemia has been traditionally characterized by increased triglycerides, increased total and LDL cholesterol, and decreased HDL cholesterol [45]. However, these lipids represent only a small subset of those in plasma [46]. By profiling 113 plasma lipids, we identified alterations in several lipid classes including bile acids, acylcarnitines, sulfated steroids, and PUFA. Bile acids were increased in HIV patients on PI-based ART, consistent with a small previous study (n=11) [47]. Under physiological conditions, bile acids play an important role in lipid absorption and homeostasis (reviewed in [48]). At high concentrations, however, bile acids are proinflammatory and hepatotoxic [48]. Acylcarnitines were also elevated in HIV subjects. Increased serum acylcarnitines, a marker of incomplete fatty acid β-oxidation, have been reported in patients with diabetes, and metabolic, cardiovascular, and mitochondrial diseases [49-51]. In rodent models of obesity and insulin resistance, increased acylcarnitines have been linked to mitochondrial overload under conditions of metabolic stress [52,53]. Another important finding was reduced plasma sulfated steroids including DHEA-S, EpiA-S, pregnenolone sulfate, and androsterone sulfate. Reduced DHEA-S has been implicated in aging, age-related comorbidities (e.g., atherosclerosis and bone metabolism), and immune dysfunction [54]. Furthermore, previous studies found associations between low DHEA-S and progression to AIDS, as well as dyslipidemia in HIV subjects on ART [55,56]. Bioactive PUFA including AA, EPA, DPA, and DHA were also decreased in the HIV plasma metabolome. PUFA depletion has been linked to hepatic triglyceride accumulation, unfolded protein responses, and increased susceptibility to endoplasmic reticulum stress [57-59]. Collectively, these alterations show substantial overlap with those previously reported in NAFLD and NASH, (i.e., bile acids, acylcarnitines, LPC, PUFA, and DHEA-S) [27-29], raising the possibility that mechanisms underlying development of NAFLD (i.e., lipid peroxidation and mitochondrial dysfunction) may also contribute to dyslipidemia in HIV patients on ART. However, other alterations were unique to HIV patients, including decreased saturated LCFA and eicosanoids. Thus, the plasma lipidome of HIV patients on PI-based ART has overlapping, yet distinctive features compared to NAFLD/NASH.

Mapping altered metabolites to KEGG and SMPDB pathways identified alterations in immunomodulatory pathways including tryptophan catabolism and eicosanoid biosynthesis (Figure 3). Despite long-term ART, tryptophan betaine and indoleproprionate were decreased and kynurenine and K:T ratios (an index of indolamine 2,3-dioxygenase (IDO) activity and tryptophan catabolism) were increased in HIV subjects compared to controls. Tryptophan catabolism is regulated by the balanced expression of IDO/tryptophanyl-tRNA-synthetase (TTS) [60,61]. Chronic IDO activation, however, drives immune dysfunction by suppressing T cell proliferation, increasing production of toxic metabolites (kynurenine and quinolinic acid), and altering the Th17/Treg balance, and has been previously linked to dysfunctional T cell responses in HIV patients [60-62]. Another important finding was decreased eicosanoids (e.g., 5-HETE, PGB2, PGE2, and TXB2). Eicosanoids, derived from PUFA, play an important role in the initiation and resolution of inflammation (reviewed in [63]). Thus, PUFA depletion and decreased eicosanoids biosynthesis may contribute to dysregulated inflammatory responses in HIV patients on PI-based ART. Correlation analysis identified negative correlations between sulfated steroids and markers of innate immune activation (IFN-α and IL-6). Mounting evidence suggests that innate immune responses regulate cholesterol/sterol metabolism. Sulfated steroids correlated inversely with plasma IFN-α, consistent with previous studies demonstrating that type I IFNs downregulate sterol metabolism during anti-viral responses [64,65]. Sulfated steroids also correlated inversely with IL-6. Dysregulated IL-6 production plays a role in age-related conditions, such as rheumatoid arthritis [66,67], osteoporosis [68], atherosclerosis [69], and Alzheimer's disease [70,71]. Consistent with our results, IL-6 has been shown to correlate inversely with serum DHEA-S in aging populations [72]. In aging mice, dysregulated IL-6 production can be reversed by supplementing animals with DHEA-S [73]. Thus, the relationship between IL-6 and sulfated steroids in HIV patients on PI-based ART merits further study.

The FIB-4 and APRI indices represent inexpensive, accurate (overall accuracy up to 87% and 72%, respectively), and non-invasive markers of liver fibrosis in HIV-infected individuals with and without HCV co-infection [31-33,74]. Similarly, circulating YKL-40 has been shown to correlate with the severity of fibrosis in chronic HCV infection and alcoholic liver disease [75,76]. Here, we found that intermediate/high FIB-4 and APRI scores and high levels of plasma YKL-40 were associated with increased plasma bile acids in HIV subjects on PI-based ART. Consistent with these findings, elevated serum bile acids have been reported in patients with cirrhosis and chronic HCV infection and were shown to correlate with the severity of liver fibrosis [30,77]. Bile acid levels (GCA and TCA) were positively correlated with acylcarnitines, a marker of incomplete fatty acid β-oxidation and mitochondrial dysfunction [52,78]. In rat models, bile acids have been shown to inhibit mitochondrial respiration in a dose dependent manner [79-81] and to enhance the permeability of mitochondria to protons [82]. Our findings, together with those of previous studies [77,83], suggest that plasma bile acids may represent a sensitive non-invasive marker of liver dysfunction in HIV patients.

Elevated plasma LPS has been reported in alcoholic liver disease [84], NAFLD [85], and inflammatory bowel disease [86] and predicts progression to end-stage liver disease in patients with chronic HCV infection [87]. Consistent with these findings, we found plasma LPS correlated positively with markers of hepatic function (bilirubin (E,E) and bilirubin (Z,Z)). The liver plays a key role in clearing LPS from the circulation, so increased plasma LPS may reflect decreased hepatic clearance [85,87,88]. In turn, circulating LPS may promote hepatic dysfunction by increasing hepatic inflammation and fibrosis [87]. Unexpectedly, LPS, LBP, and bilirubin ((E,E) and (Z,Z)) were negatively correlated with LCFA. LCFA deficiency has been reported in patients with advanced cirrhosis and other liver diseases (acute hepatitis, cholestasis) [87,89]. Thus, decreased plasma LCFA may be indicative of hepatic dysfunction in HIV patients. Together, these findings suggest complex inter-relationships between hepatic dysfunction, microbial translocation, and dyslipidemia in HIV patients on PI-based ART.

We acknowledge certain limitations of our study. We selected HIV subjects with advanced disease at increased risk for dyslipidemia (PI-based ART) and hepatic dysfunction (HCV). Further studies are required to define metabolic alterations in cohorts who are treatment-naive, at earlier stages of disease, on specific treatment regimens, with acute vs. chronic HCV infection, and with clinical evidence of metabolic abnormalities such as lipodystrophy and insulin resistance. Importantly, >60% of signature metabolites altered in HIV subjects on PI-based ART were also altered in an independent cohort of HIV subjects on NNRTI-based ART including sulfated steroids, bile acids, and LCFA (Additional file 2), indicating that many of the metabolite alterations we detected in HIV subjects on PI-based ART are not unique to this class of ART drugs. Future studies are required to better understand which metabolic abnormalities are associated with ART versus HIV infection. Individuals in both cohorts were predominantly African American. African Americans have higher rates of coronary heart disease and metabolic syndrome compared to Caucasians [90]. Thus, further studies are needed to investigate these findings in other races/ethnicities. Finally, our study cohort was relatively small. Nonetheless, there was sufficient statistical power to identify metabolite sets with significant differences between HIV subjects with advanced disease and healthy controls. Despite these limitations, by applying stringent selection criteria, our study identified significant alterations in several important lipid classes associated with markers of inflammation and hepatic function in HIV patients on PI-based ART.

In summary, metabolomic profiling identified alterations in the plasma lipidome of HIV patients on PI-based ART, including increased bile acids and acylcarnitines and decreased sulfated steroids, PUFA, and LPC. Correlation analysis identified alterations in distinct lipid classes linked to markers of inflammation, microbial translocation, and hepatic function. Future studies are required to determine if these correlations reflect direct or indirect interactions and define the mechanisms underlying these inter-relationships. Many of the altered lipids represent important ligands of the nuclear receptor superfamily. Bile acids are endogenous ligands for FXR, which regulates bile acid biosynthesis and hepatic fatty acid and triglyceride biosynthesis [91]. In mice, FXR activation protects against ritonavir-induced dyslipidemia and aortic plaque development, reverses insulin resistance and lipid abnormalities, and protects against hepatic steatosis [92,93]. Sulfated steroids and bile acids are ligands for PXR and CAR, which regulate expression of Phase I and II drug-metabolizing enzymes and transporters and hepatic lipid metabolism [91]. Omega-3 and omega-6 PUFA modulate transcription via interactions with PPARs, which increase fatty acid oxidation and suppress lipogenesis and control inflammation by transrepression of NF-κB, NFAT, AP-1, and STATs [39,91]. Clinical trials have only shown modest effects of PPARα (fibrates) and PPARγ (thiazolidinediones and pioglitazone) agonists in reversing ART lipoatrophy [94,95], while other studies suggested these drugs may be useful in treating ART-associated diabetes and NAFLD [96].

## Conclusions

By untargeted metabolomic profiling, this study provides better understanding of metabolic abnormalities in HIV patients with advanced disease on PI-based ART. Lipid abnormalities were linked to markers of inflammation, microbial translocation, and hepatic function, suggesting that therapeutic strategies attenuating innate immune activation and hepatic dysfunction may offer new approaches for prevention and treatment of dyslipidemia on PI-based regimens. Conversely, reversing lipid abnormalities in HIV patients may be beneficial for attenuating inflammation, immune dysfunction, and hepatic dysfunction. Plasma bile acids may represent a sensitive non-invasive marker of liver dysfunction in HIV patients on ART. Untargeted metabolomic profiling represents a powerful tool for gaining a systems-level understanding of metabolic alterations and their relationship to dysfunction involving other organ systems that will be important for developing therapeutic strategies targeting metabolic disease and chronic inflammation in HIV patients on ART.

## Competing interests

The authors declare that they have no competing interests.

## Authors’ contributions

EC performed the experiments and data analysis, drafted the manuscript, and prepared figures. VM performed bioinformatics and statistical analysis and prepared figures. AH and AK carried out part of the data analysis and contributed to study design and bioinformatics. SM participated in study design, data analysis, and manuscript editing. DG conceived of the study, participated in its design, coordination, and data analysis, and helped write and edit the manuscript. All authors read, participated in editing the manuscript, and approved the final manuscript.

## Pre-publication history

The pre-publication history for this paper can be accessed here:

http://www.biomedcentral.com/1471-2334/13/203/prepub

## Supplementary Material

Additional file 1 **Alterations in the plasma metabolome of HIV patients on PI-based ART compared to healthy controls.** Shown is 682 the total dataset of 227 metabolites detected in the initial and validation 683 cohorts following data pre-processing. Significantly altered metabolites 684 were defined by a fold change (FC) >1.3, p-value <0.05, and FDR ≤0.1. Click here for file

Additional file 2 **Majority of signature metabolites are altered in an independent cohort of HIV subjects on NNRTI-based ART.** An independent 674 cohort of HIV subjects on NNRTI-based ART (n=8) was matched to HIV 675 subjects in initial and validation cohorts by age, gender, HCV serostatus, 676 and stage of HIV disease (nadir and current CD4 count). Significantly 677 altered metabolites were defined by a FC >1.3, p-value ≤0.1 and FDR 678 ≤0.1 compared to healthy controls (n=8) matched for age, gender, and 679 race/ethnicity. Click here for file

Additional file 3 **Sub-analysis of HIV plasma metabolome according to HCV serostatus.** Analysis was performed on a subset of HIV subjects with (n=10) and without HCV co-infection (n=7) matched for age, gender, and race/ethnicity. Shown is the total dataset of 227 metabolites detected in the initial and validation cohorts following data pre-processing. Significantly altered metabolites were defined by a FC >1.3, p-value ≤0.1 and FDR ≤0.1 compared to healthy controls (n=8) matched for age, gender, and race/ethnicity. Click here for file

Additional file 4 **Alterations in the plasma lipidome of all HIV subjects compared to controls.** Shown are 113 lipids detected in the merged dataset of all HIV subjects on PI-based ART (n=32) compared to healthy controls (n=20). Significantly altered metabolites were defined by a FC >1.3, p-value <0.05 and FDR ≤0.1. Click here for file

Additional file 5 **Pearson correlation analysis of lipid metabolites detected in plasma from HIV patients on PI-based ART.** Pearson correlation analysis was performed in R using cor.test function. FDR were generated in R using fdrtool. Shown are correlation coefficients (p-values) (FDR). Click here for file

Additional file 6 **Lipid metabolites detected in plasma from HIV patients on PI-based ART and HIV-negative individuals with NAFLD/NASH.** NAFLD and NASH datasets were based on results from 3 previously published studies [36-38]. Red metabolites were altered in HIV patients compared to healthy controls in the present study or were previously reported to be altered in patients with NAFLD or NASH compared to healthy controls. Click here for file
